# Identification of Prognostic Factors in Cholangiocarcinoma Based on Integrated ceRNA Network Analysis

**DOI:** 10.1155/2022/7102736

**Published:** 2022-09-15

**Authors:** Haili Jin, Wei Liu, Weiming Xu, Liping Zhou, Huarong Luo, Cheng Xu, Xi Chen, Wenbin Chen

**Affiliations:** ^1^Pathology Department, Taizhou Hospital, Zhejiang University, Taizhou, 317000 Zhejiang, China; ^2^Emergency Department, Taizhou Hospital of Zhejiang Province Affiliated to Wenzhou Medical University, Taizhou, 317000 Zhejiang, China; ^3^Emergency Department, Enze Hospital, Taizhou Enze Medical Center (Group), Taizhou, 318050 Zhejiang, China; ^4^Pathology Department, Taizhou Hospital of Zhejiang Province Affiliated to Wenzhou Medical University, Taizhou, 317000 Zhejiang, China; ^5^Pathology Department, Enze Hospital, Taizhou Enze Medical Center (Group), Taizhou, 318050 Zhejiang, China; ^6^Chemical Pharmaceutical Research Institute, Taizhou Vocational & Technical College, Taizhou 318000, China; ^7^Department of Colorectal Surgery, The First Affiliated Hospital, Zhejiang University School of Medicine, Hangzhou, 310003 Zhejiang, China

## Abstract

This study is aimed at screening prognostic biomarkers in cholangiocarcinoma (CHOL) based on competitive endogenous RNA (ceRNA) regulatory network analysis. Microarray data for lncRNAs, mRNA, and miRNAs were downloaded from the GEO and TCGA databases. Differentially expressed RNAs (DERs) were identified in CHOL and normal liver tissue samples. WGCNA was used to identify disease-related gene modules. By integrating the information from the starBase and DIANA-LncBasev2 databases, we constructed a ceRNA network. Survival analysis was performed, and a prognostic gene-based prognostic score (PS) model was generated. The correlation between gene expression and immune cell infiltration or immune-related feature genes was analyzed using TIMER. Finally, real-time quantitative PCR (RT-qPCR) was used to verify the expression of the 10 DERs with independent prognosis. A large cohort of DERs was identified in the CHOL and control samples. The ceRNA network consisted of 6 lncRNAs, 2 miRNAs, 90 mRNAs, and 98 nodes. Ten genes were identified as prognosis-related genes, and a ten-gene signature PS model was constructed, which exhibited a good prognosis predictive ability for risk assessment of CHOL patients (AUC value = 0.975). Four genes, ELF4, AGXT, ABCG2, and LDHD, were associated with immune cell infiltration and closely correlated with immune-related feature genes (CD14, CD163, CD33, etc.) in CHOL. Additionally, the consistency rate of the RT-qPCR results and bioinformatics analysis was 80%, implying a relatively high reliability of the bioinformatic analysis results. Our findings suggest that the ten-signature gene PS model has significant prognostic predictive value for patients with CHOL. These four immune-related DERs are involved in the progression of CHOL and may be useful prognostic biomarkers for CHOLs.

## 1. Introduction

Cholangiocarcinoma (CHOL) is an aggressive epithelial malignancy that frequently arises from cholangiocytes or the biliary tract. It is the second most common liver cancer after hepatocellular carcinoma (HCC) worldwide and accounts for approximately 3% of gastrointestinal tumors [1]. The incidence of CHOL is 0.3-6/100,000 individuals per year, and mortality is 1-6/100,000 individuals per year [2, 3]. However, the rate has been increasing in most countries in recent years, particularly in some Southeast Asian areas [4]. CHOL is usually asymptomatic in the early stages, and patients are often diagnosed at advanced stages, leading to a dismal prognosis [5]. Therefore, it is critical to develop effective biomarkers and molecules for its early detection and treatment.

The competing endogenous RNA (ceRNA) is a novel regulatory network that has been proven to be a major factor in cancer development. The regulatory patterns and crosstalk between miRNAs, lncRNAs, and mRNA have been widely uncovered in recent studies [6, 7]. In addition, lncRNAs act as ceRNAs and regulate a series of miRNA activities by sponging these miRNAs and are involved in the regulation of posttranscriptional processes [8, 9]. However, little is known regarding the ceRNA regulatory mechanisms in CHOL. Bioinformatics tools and multiomics analysis have enabled effective data mining to understand the molecular mechanism of this cancer. By systematically analyzing whole-transcriptome sequencing data, Chu et al. identified many differentially expressed RNAs associated with CHOL, and *miR-144-3p* plays a fundamental role in CHOL genesis [10]. Based on the expression profile analysis of ceRNA, Wang et al. constructed an lncRNA-miRNA-mRNA network and screened several prognosis-related lncRNAs, such as *COL18A1-AS1* and *SLC6A1-AS1* [11]. The potential role of upstream lncRNAs was also explored. A recent study demonstrated *lncRNA TTN-AS1* promotes CHOL progression through *miR-320a*/neuropilin-1 axis [12]. There is insufficient ceRNA data profiling of potential biomarkers for CHOL diagnosis and prognosis.

In this study, we downloaded microarray datasets from public databases and analyzed the expression levels of miRNAs, lncRNAs, and mRNA between CHOL and normal tissue samples. The optimal pairwise gene was screened to construct a ceRNA regulatory network, along with survival analysis. We established ten signature genes to predict the prognosis of patients with CHOL. Finally, the correlation between prognostic genes and immune cell infiltration was analyzed to identify immune-related genes. These findings proved that the ten-gene signature model can be applied to predict the prognosis of CHOLs.

## 2. Materials and Methods

### 2.1. Data Mining from Public Database

Microarray datasets were derived from the GEO database in May 2019 by setting “cholangiocarcinoma, homo sapiens” as the keywords. The screening criteria were as follows: the dataset should be whole-genome sequencing profiles, including CHOL and normal tissue samples, and the sample number should be more than 100. Finally, we obtained the GSE26566 dataset [13], which included 104 CHOL and 59 normal perihepatic tissue samples. Profiles were tested on an Illumina Humanref-8 V2.0 expression beadchip platform.

The mRNA and miRNA samples associated with CHOL were downloaded from The Cancer Genome Atlas database (TCGA, https://gdc-Portal.nci.nih. gov/). After corresponding barcode information to the samples, we screened 45 samples with matched mRNA and miRNA, including nine normal samples and 36 CHOL tumor samples.

In addition, the mRNA and miRNA profiles of HCC were downloaded from TCGA and utilized as validation sets, which consisted of 424 and 420 samples, respectively. After matching the barcode information to the samples, we obtained 358 paired mRNA and miRNA tumor samples, which were used as auxiliary verification samples after constructing the model.

### 2.2. Differential Expressed Analysis

First, the expression levels of ceRNAs in TCGA samples were reannotated according to the corresponding information. We extracted the annotation profiles of GSE26566 from the Ensembl genome browser 96 (http://asia.ensembl.org/index.html), including probes, gene symbols, and RNA types. The Limma package [14] (Version 3.34.0) was used to screen differentially expressed RNAs from TCGA and GSE26566 datasets by comparing the CHOL and control samples. FDR value < 0.05 and |log2 fold change (FC)| > 1 were set as thresholds.

Hierarchical clustering analysis was performed using the pheatmap package [15] (version 1.0.8, https://cran.cran.project.org/package=pheatmap), and a heatmap was generated based on the Encyclopedia of Distance [16] and the expression level of DERs. Finally, we selected the overlapping genes between TCGA and GSE26566 datasets for further analysis.

### 2.3. Weighted Gene Correlation Network Analysis

The WGCNA package [17, 18] (version 1.61) was used to screen for stable gene modules associated with CHOL. We applied TCGA dataset as the training set and GSE26566 profiles as the testing set. Correlation analysis of gene expression was conducted between the two datasets, followed by steps of adjacency function definition, gene module division, assessment of module stability, functional annotation of stable modules, and correlation analysis of clinical parameters. The screening threshold was cutHeight = 0.99, and the gene modules contained more than 30 genes.

### 2.4. ceRNA Regulatory Network Construction

We researched the DIANA-LncBasev2 database [19] (http://carolina.imis.athena-innovation.gr/diana_tools/web/index.php?(r= lncbasEV2% findex-exp ErimENTAL) to screen DElncRNA and DEmiRNA pairs, and only the pairwise gene with opposite expression status was conserved for ceRNA network construction.

In addition, we researched the conversed miRNA target site to characterize predictive target genes according to starBase [20] (version 2.0, http://starbase.sysu.edu.cn/), which provides five algorithms (TargetScan, PicTar, RNA22, PITA, and miRanda) to explore miRNA-mRNA interaction maps from CLIP-sequencing data.

By integrating the DERs screened in previous steps, we constructed a ceRNA regulatory network and visualized the connection of genes using Cytoscape 3.6.1 software [21]. Functional analysis was performed using DAVID version 6.8 [22]. A *P* value less than 0.05 was set as the threshold to screen critical biological terms and signaling pathways.

### 2.5. A Prognostic Predictive Model

For these ceRNAs in the regulatory network, we conducted univariate and multivariate Cox regression analyses using the survival package [23] (version 2.41-1, http://bioconductor.org/packages/survivalr/). Prognosis-related DERs (including lncRNA, miRNA, and mRNA) were screened based on prognosis information (such as age, gender, pathological TNM, and tumor grade) of 36 CHOL samples in TCGA. A log-rank *P* value less than 0.05 was selected as a threshold. In addition, we developed a prognostic score (PS) model for prognosis prediction based on the prognostic coefficient value of each RNA and expression value.

(1) $$\begin{array}{c} \text{Prognostic score }\left(\text{PS}\right)=\sum \beta_{\text{RNAs}}\times {\text{Exp}}_{\text{RNAs}}. \end{array}$$

In this study, *β*_RNAs_ represented the prognostic coefficient of each RNAs in multivariable analysis, and Exp_RNAs_ represented the expression level of RNAs in the training sets.

All individuals in the TCGA training set can be divided into high- and low-risk groups based on the threshold of the median PS value. The predictive ability of the PS model was validated using Kaplan-Meier curve analysis and receiver-operator characteristic (ROC) curve analysis.

### 2.6. Correlation of Gene Expression and Immune Cell Infiltrating/Immune-Related Feature Genes

The correlation between target gene expression and the infiltration of immune cells or immune-related feature genes (CD14, CD163, CD19, CD22, CD24, CD27, CD33, CD34, CD38, CD4, CD44, CD5, CD80, D86, FOXP3, KIT, and TLR2) was analyzed using the online tool TIMER [24, 25], which is a comprehensive resource for systemic analysis of immune infiltration across various cancer types.

### 2.7. Real-Time Quantitative PCR (RT-qPCR) Assay

The obtained ten DERs with independent prognosis correlations, including eight mRNAs, one lncRNA, and one miRNA, were selected for RT-qPCR verification. Two cell lines, normal human intrahepatic bile duct epithelial cells (HIBEC) and cholangiocarcinoma cells (HuCCT1), were purchased from Shanghai Fuheng Biotechnology Co., Ltd. (Shanghai, China). They were maintained in Roswell Park Memorial Institute 1640 (RPMI-1640, Thermo Fisher Scientific, Waltham, MA USA), supplemented with 10% fetal bovine serum (FBS, Thermo Fisher Scientific), and cultured in an incubator with 5% carbon dioxide at 37°C.

After two passages, the cells were harvested to extract total RNA using RNAiso Plus reagent (TAKARA, Shiga, Japan), following the manufacturer's instructions. Then, the PrimeScript™ II 1st Strand cDNA synthesis Kit (TAKARA Biomedical Technology Co., Ltd., Beijing, China) was used to reverse transcribe total RNA to cDNA according to the manufacturer's protocols. The level of miR-25-3p was measured using the stem-loop method, with U6 serving as a reference gene. For the expression of lncRNA MIR99AHG and the eight mRNAs, GAPDH was used as the housekeeping gene. The sequences of all primers are listed in Supplementary Table S1. The RT-qPCR reaction was shown as follows: 50°C for 2 min, 95°C for 2 min, a total of 40 cycles at 95°C for 15 s and 60°C for 60 s, 95°C for 15 s, 60°C for 1 min, and 95°C for 15 s. The relative expression levels of the lncRNAs, miRNAs, and mRNAs were calculated using the 2^–ΔΔCT^ method.

### 2.8. Statistical Analysis

Data are expressed as mean ± standard deviation. GraphPad Prism 8 (San Diego, CA, USA) was used for statistical analysis. The Student's *t*-test was used for comparative analysis between the two groups. *P* < 0.05 was considered as the statistical significance.

## 3. Results

### 3.1. Screening Differentially Expressed RNAs

**Figure 1:**
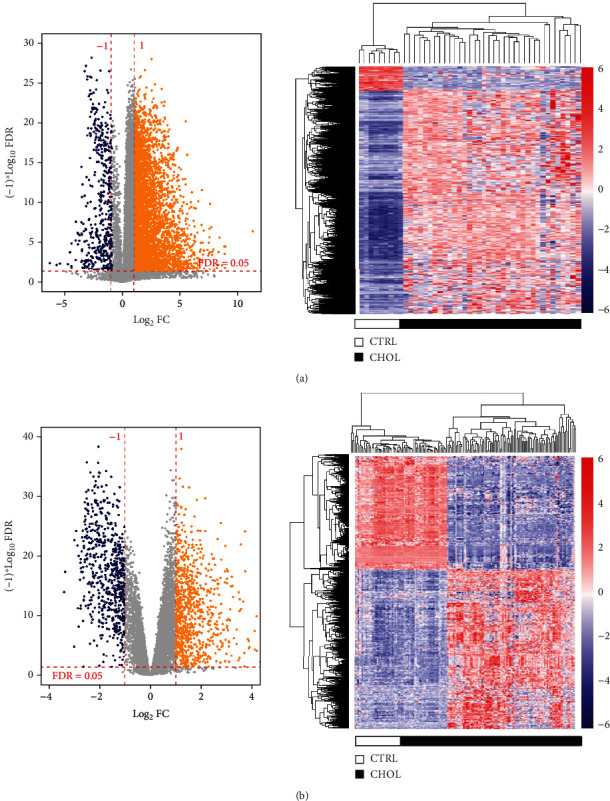
Volcano plots and clustering analysis of differential expressed RNAs in TCGA (a) and GSE26566 (b) datasets. Blue dots represent the dysregulated genes. Red horizontal line indicates FDR < 0.05, while vertical dotted lines denote |log2FC| > 1.

After data preprocessing, we screened 4675 and 1521 DERs from TCGA and GSE26566 datasets, respectively, using the criteria of FDR value < 0.05 and |log2FC| > 1. Volcano plots and hierarchical clustering heatmaps are shown in Figure 1. The differential expression of genes could help distinguish CHOL tissues from normal liver tissues based on clustering analysis. By comparing the two sets of DERs, we obtained 1031 overlapping RNAs. A total of 1008 DERs with consistent expression levels were considered critical genes.

### 3.2. Identification of Disease-Related Modules by WGCNA

**Figure 2:**
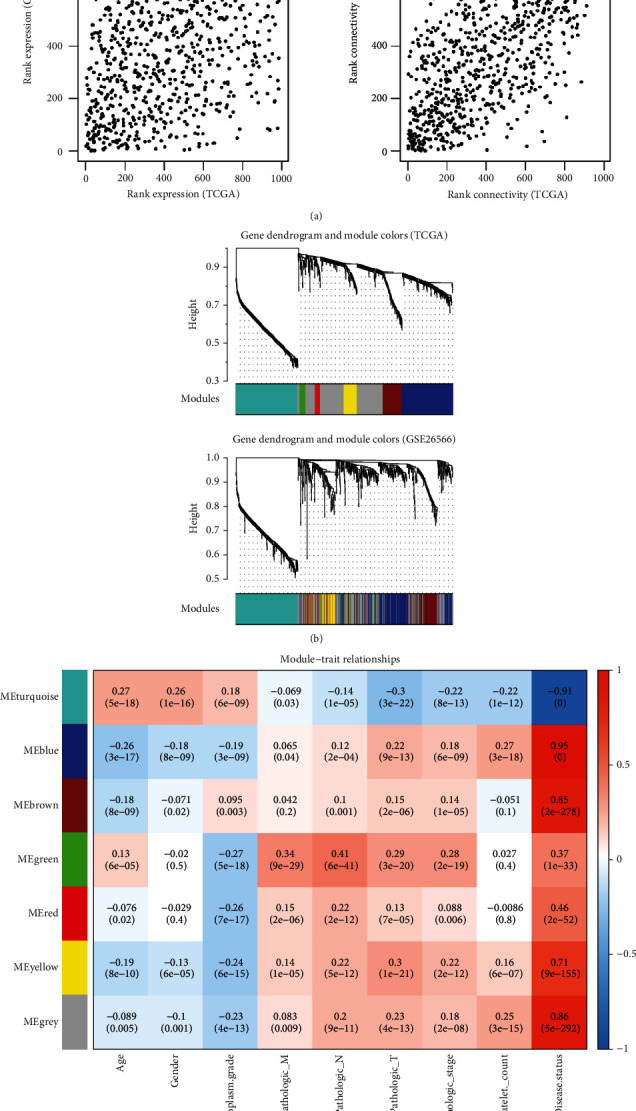
Weighted gene correlation network analysis to screen gene modules. (a). Gene coexpression analysis and network connectivity analysis. (b). Clustering dendrogram of CHOL-related genes based on TCGA and GSE26566 dataset. (c). Heatmap of modules and clinical trait relationships. The color changes from blue to red represent the changes from negative correlation to positive correlation.

First, the correlations of gene expression between the two datasets and network connectivity correlations were analyzed. The power value was set to 12, and the median connectivity of genes satisfied the scale-free network (Figure 2(a)).

When cutHeight = 0.99, we identified seven gene modules named modules 1-7, and the corresponding colors were blue, brown, green, grey, red, turquoise, and yellow (Table 1). Heatmaps were generated to visualize the correlation between the functional modules and clinical traits (Figure 2(b)). We identified four gene modules as stable functional modules based on the threshold of preservation *Z* score > 5 and *P* value < 0.05, including modules 1, 2, 6, and 7 (Figure 2(c)). Therefore, 663 genes in the four modules were considered candidate genes for further analysis.

### 3.3. Creation of ceRNA Regulatory Network for Candidate Genes in CHOL

Using the predictive tool LncBasev2, we identified several pairwise lncRNAs and miRNAs. Thus, six gene pairs with opposite expression statuses were considered potential genes related to disease progression. Using starBase, we researched the target genes regulated by DERs of the four modules and finally obtained 90 gene pairs with negative correlation at the gene expression level.

**Figure 3:**
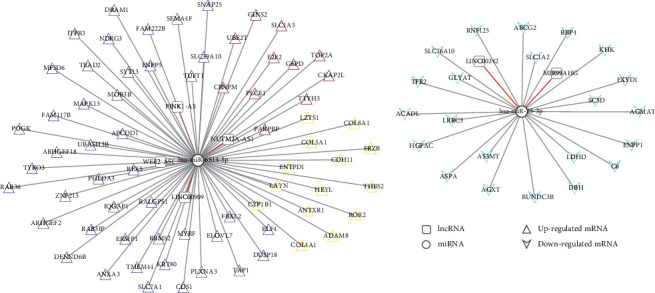
CeRNA regulatory network construction. Square and circle were differential expressed lncRNA and miRNA. Equilateral triangle and inverse triangle represent up- and downregulated genes. The node with differential color was corresponded to related-module color.

**Figure 4:**
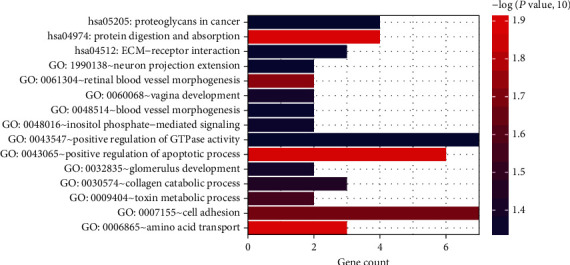
GO function and KEGG pathway analysis for ceRNA network-based differential expressed RNAs. The horizontal axis refers to gene number, whereas vertical axis means items of biological process or pathways. Increased intensity of red color means more significantly enriched genes.

In addition, we constructed a ceRNA regulatory network composed of 6 lncRNAs, 2 miRNAs, 90 mRNAs, and 98 nodes (Figure 3). The two miRNAs (hsa-miR-25, hsa-miR-6514-5p) were significantly enriched in the ceRNA complex network, which also included 68 upregulated and 22 downregulated mRNAs. Functional analysis showed that these DERs were mainly enriched in 13 biological processes and three signaling pathways (Figure 4, Table 2), including cell adhesion, positive regulation of GTPase activity, ECM-receptor interaction, protein digestion and absorption, positive regulation of apoptotic process, and proteoglycans in cancer.

### 3.4. Development of Prognostic Score Model

The correlation between clinical features and prognosis of CHOL patients was analyzed using univariate and multivariate Cox regression analyses. It was found that in the univariate Cox regression analysis, only prognostic score status was significantly related to the prognosis of CHOL (*P* = 5.65*E* − 04), whereas in the multivariate Cox regression analysis, the prognosis of CHOL was significantly associated with age (*P* = 4.65*E* − 02) and prognostic score status (*P* = 1.91*E* − 02, Table 3).

To further identify prognosis-related genes in the ceRNA network, we also conducted univariate and multivariate Cox regression analyses for these DERs. As a result, ten DERs with independent prognostic correlations were obtained, including eight mRNA, one lncRNA, and one miRNA (Table 4).

**Figure 5:**
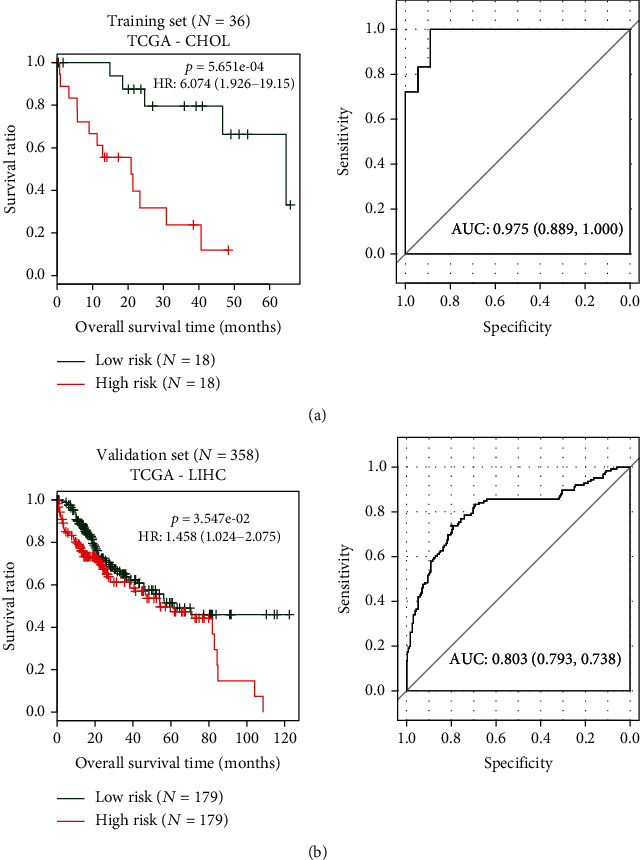
Receiver-operating characteristic (ROC) curve for CHOL patients in training and validation set based on prognostic score model. Green and red curves represent patients in low-risk and high-risk groups, respectively.

A ten-gene signature PS model was constructed according to the coefficient and expression values of the DERs. The predictive ability of the PS model was validated using TCGA and HCC validation sets. Samples in the two datasets could be divided into high- and low-risk groups based on the threshold of the median PS value. Survival analysis revealed that CHOL or HCC patients in the high-risk group exhibited a poor prognosis (Figure 5; *P* < 0.001, HR: 6.074 [1.926-19.15]; *P* < 0.05, HR: 1.458 [1.024-2.075]), which was consistent with the actual disease status. The area under the curve (AUC) was 0.975, indicating good predictive ability for the risk assessment system in patients with CHOL.

### 3.5. Correlation Analysis of Critical Genes and Tumor-Infiltrating Immune Cells/Immune-Related Feature Genes

**Figure 6:**
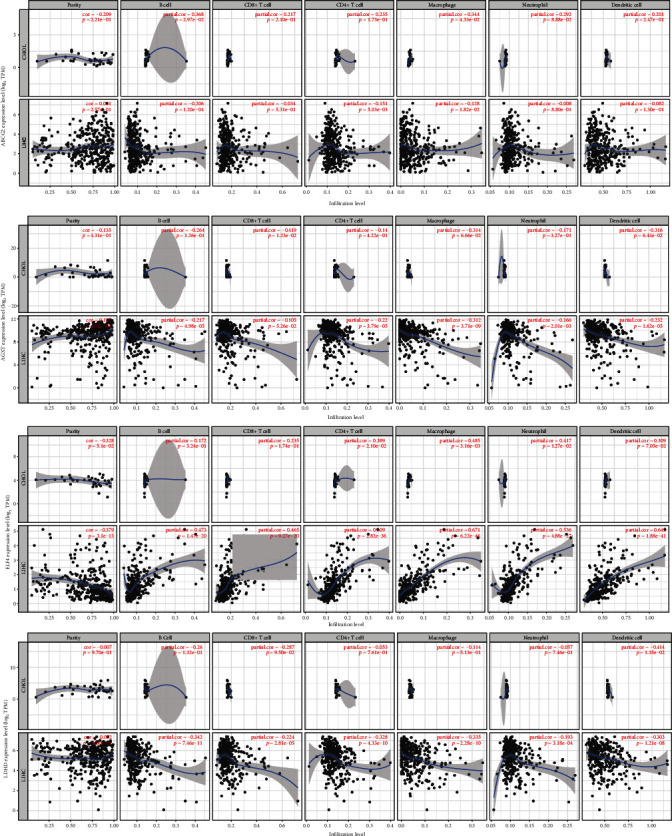
Correlation analysis of prognostic genes and immune cell infiltration. Scatter plots were generated with Spearman's correlation and statistical significance. *ELF4* were correlated with immune infiltration of CD4+ cell, CD8+ cell, and neutrophil (*P* < 0.05).

The correlation between tumor-infiltrating immune cells and target gene expression was analyzed for eight DEGs. The results showed that most genes were significantly correlated with tumor-infiltrating immune cells in HCC samples (Figure 6). However, only four genes (ABCG2, AGXT, ELF4, and LDHD) were associated with immune cell infiltration in CHOL patients. For example, the dysregulation of *ELF4* was correlated with CD4^+^ cells (*P* < 0.05), CD8^+^ cells (*P* < 0.05), and neutrophil infiltration (*P* < 0.05). *AGXT* was correlated with CD8^+^ cell infiltration (*P* < 0.05). Our results indicate that these four genes might be prognostic immune-related genes in CHOL progression.

**Figure 7:**
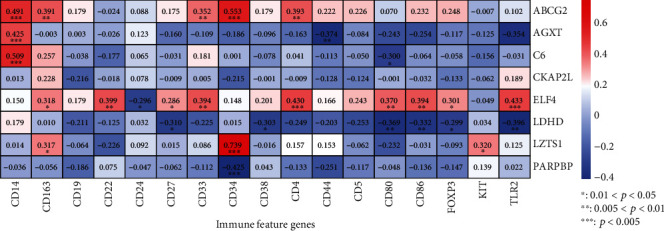
Correlation analysis of the eight prognostic genes and immune-related feature genes. A heatmap was generated with Spearman's correlation and statistical significance. ^∗^: 0.01 < *P* < 0.05; ^∗∗^: 0.005 < *P* < 0.01; ^∗∗∗^: *P* < 0.005. Red represented positive correlation, and blue represented negative correlation. The darker the color, the more significant the correlation.

In addition, the correlation between immune-related feature genes and the expression of the eight DEGs was also analyzed. As shown in Figure 7, *ABCG2* was positively correlated with *CD14*, *CD163*, *CD33*, *CD34*, and *CD4* (*P* < 0.05); *AGXT* and *C6* were positively correlated with the expression of *CD14* (*P* < 0.05), while it was negatively correlated with *CD44* and *CD80* (*P* < 0.05). *CKAP2L* expression was not significantly correlated with these immune-related genes (*P* > 0.05). For *ELF4*, it was positively correlated with *CD163*, *CD22*, *CD27*, *CD33*, *CD4*, *CD80*, *CD86*, *FOXP3*, and *TLR2* (*P* < 0.05), whereas it was negatively correlated with *CD24* (*P* < 0.05). *LDHD* was negatively correlated with *CD27*, *CD38*, *CD80*, *CD86*, *FOXP3*, and *TLR2* (*P* < 0.05), while *LZTS1* had positive correlation with *CD163*, *CD34*, and *KIT* (*P* < 0.05). Furthermore, *PARPBP* expression was negatively related with the expression of *CD34* (*P* < 0.05).

### 3.6. Verification of RT-qPCR

**Figure 8:**
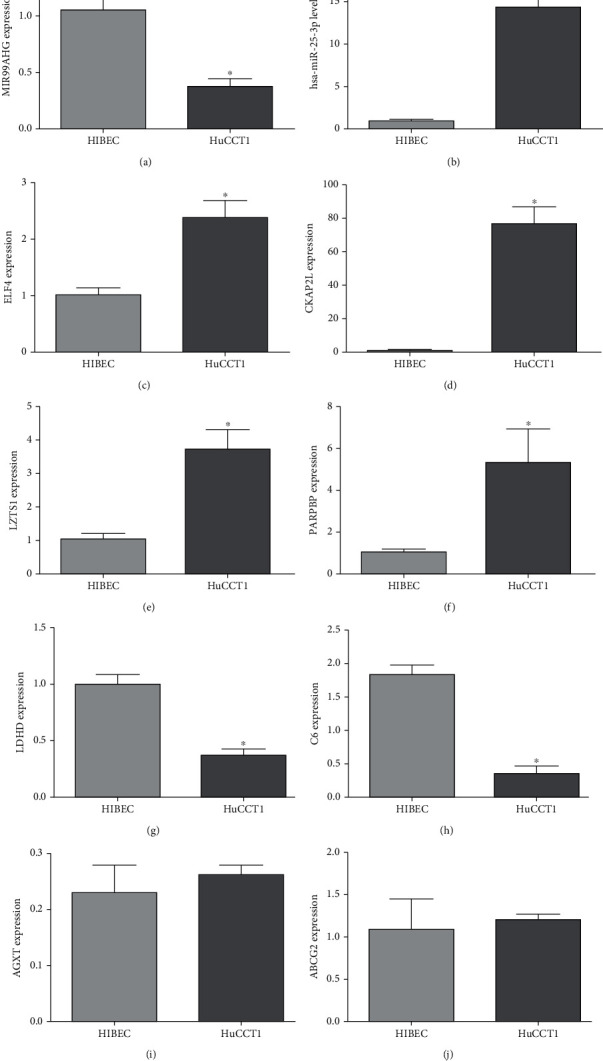
Verification of the ten DERs with independent prognosis correlation, including 8 mRNAs, 1 lncRNA, and 1 miRNA in HIBEC cells and HuCCT1 cells using real-time quantitative PCR. The expression levels of lncRNA MIR99AHG (a), hsa-miR-25-3p (b), ELF (c), CKAP2L (d), LZTS1 (e), PARPBP (f), LDHD (g), C6 (h), AGXT (i), and ABCG2 (j). ^∗^: *P* < 0.05, compared with the HIBEC cells.

Ten DERs with independent prognostic correlation, including five upregulated (miR-25-3p, *ELF4*, *CKAP2L*, *LZTS1*, and *PARPBP*) and five downregulated (lncRNA MIR99AHG, *LDHD*, *C6*, *AGXT*, and *ABCG2*) DERs, were chosen for RT-qPCR verification. It was found that the lncRNA MIR99AHG expression level was significantly lower in HuCCT1 cells than in HIBEC cells (*P* < 0.05, Figure 8(a)), and the miR-25-3p level in HuCCT1 cells was significantly higher than that in HIBEC cells (*P* < 0.05, Figure 8(b)). For the remaining eight mRNAs, compared with the HIBEC cells, the expression levels of ELF4, CKAP2L, LZTS1, and PARPBP were evidently upregulated in HuCCT1 cells (*P* < 0.05), whereas the expression of LDHD and C6 was evidently downregulated (*P* < 0.05, Figures 8(c)–8(h)), which was in line with the expression patterns of the results of bioinformatics analysis. However, there was no significant difference in the expression of *AGXT* and *ABCG2* between HIBEC and HuCCT1 cells (*P* > 0.05, Figures 8(i) and 8(j)). All results showed that the consistency rate of RT-qPCR results and bioinformatics analysis was 80% (8/10), implying a relatively high reliability of the bioinformatics analysis results.

## 4. Discussion

In this study, we screened a large number of DEGs, DELs, and DEMs between CHOL and normal liver samples. These DERs are associated with cell adhesion, positive regulation of GTPase activity, ECM-receptor interactions, and proteoglycans in cancer. Survival analysis results revealed ten-hub genes were associated with CHOL prognosis. We constructed a ten-gene-based model and validated its prognostic predictive ability. In addition, RT-qPCR was used to verify the expression of the ten prognosis-related DERs and showed relatively high reliability of the bioinformatics analysis results.

In the ceRNA regulatory network, *hsa-miR-6514-5p* and *hsa-miR-25-3p* could interact with multiple genes and lncRNAs. MiRNAs are important components of gene regulation. miR-25 belongs to the miR-106b~25 family. Two studies have reported *miR-25* is upregulated in CHOL tissue samples and cancer cell lines [26, 27]. Functionally, *miR-25* could promote apoptosis resistance in CHOL cells by targeting TRAIL death receptor-4. The high expression level of *miR-25* was significantly correlated with TNM stage, lymph node metastasis, and unfavorable prognosis in CHOLs [27]. LncRNA-MIR99AHG or MONC produces a spliced lncRNA and three miRNAs: *mi99A*, *mi125B2*, and *LET7C*. Previous studies showed *lncRNAs-MIR99AHG* was an oncogene upregulated in patients with acute megakaryoblastic leukemia and gastric cancer [28, 29]. It functions as a ceRNA of *miR-577* and thus inhibits cancer cell apoptosis by activating the Wnt/*β*-catenin pathway [29]. Dysregulation of *MIR99AHG* is correlated with unfavorable survival times in patients with various cancer types [30, 31]. Our results are consistent with those of previous studies. Furthermore, our RT-qPCR results showed that compared with normal cells, lncRNA MIR99AHG expression was downregulated, whereas miR-25-3p was upregulated in HuCCT1 cells. Therefore, we speculated that MIR99AHG may act as a ceRNA of tumour-miR-25-3p to regulate target genes, thus playing critical roles in the progression of CHOL.

In addition, we constructed a ten-gene signature model to predict the prognosis of CHOLs, including lncRNA MIR99AHtumourhsa-miR-25-3p, and eight DEMs: *ABCG2*, *AGXT*, *ELF4*, *LDHD*, *C6*, *CKAP2L*, *LZTS1*, and *PARPBP*. RT-qPCR results showed that *ELF4*, *CKAP2L*, *LZTS1*, and *PARPBP* were upregulated, whereas *LDHD* and *C6* were downregulated in the HuCCT1 cells. No significant differences were observed in the expression of *AGXT* and *ABCG2* between HIBEC and HuCCT1 cells. ABCG2 mediates the transport of various substances in cellular processes. The expression of *ABCG2* has been implicated in multiple drug resistance and poor prognosis in several cancer types. Subcellular localization of *ABCG2* transporter plays a protective role in normal gallbladder epithelial cells; cellular accumulation of *ABCG2* in poorly differentiated cancer might correlate with the activation of PI3K signaling pathways [32]. Downregulation *ABCG2* might promote tumor progression and contribute to the aggressive growth of CHOL [33]. High *ELF4* expression in human cancers is associated with worse disease outcomes and increased resistance to anticancer drugs [34]. CKAP2L, an independent risk factor, is closely related to glioma prognosis [35]. In HCC, dysregulation of *PARPBP* [36] and *AGXT* [37] was also shown to be correlated with patient prognosis. A previous study showed that the downregulation of *LDHD* expression could be a predictor of poor prognosis in patients with clear cell renal cell carcinoma [38]. *LZTS1* inhibits HCC cell proliferation by suppressing the PI3K/Akt pathway [39]. In summary, it can be inferred that our ten-gene model may be used as a potentially reliable method for prognosis prediction of cancer patients, and upregulation of hsa-miR-25-3p, *ELF4*, *CKAP2L*, *LZTS1*, and *PARPBP*, and downregulation of lncRNA MIR99AHG, *LDHD*, and *C6* may be prognostic biomarkers of CHOL development. However, the specific roles of these prognostic genes in CHOL progression require further investigation.

Furthermore, *ABCG2*, *ELF4*, *LDHD*, and *AGXT* mRNA expression was significantly related to immune cell infiltrating and immune-related feature genes (such as CD14, CD63, CD33, FOXP3, and TLR2) by Person's correlation analysis. Tumor microenvironment (TME) consists tumor cells, surrounding stroma, and various infiltrating immune cells, which contributed to tumor heterogeneity [40]. Tumor-infiltrating immune cells are correlated with survival outcomes and disease-free survival in various cancer types [41, 42]. Notably, we identified that the expression of *ELF4* was significantly correlated with CD4^+^ cells, CD8^+^ cells, and neutrophils. In addition, ELF4 was positively correlated with CD163, CD22, CD27, CD33, CD4, CD80, CD86, FOXP3, and TLR2 while negatively correlated with CD24. *ELF4* or myeloid *ELF1*-like factor (MEF) is a member of the ETS family. The general feature of *ELF4* is critically associated with cellular processes, such as immune response, osteogenesis, and cancer cell quiescence [43]. Yamada et al. [44] reported *ELF4* controls CD8^+^ T cell homing and proliferation through the tumor suppressors KLF4 and KLF2. *ELF4^–/–^* mice displayed accumulated CD8^+^ T cells during steady-state conditions, an increased memory T cell population after immunization, and redistribution of T cells to non-lymphatic tissues, indicating the important role of ELF4 in immunity. Transcript fusion of *BCORL1-ELF4* has been identified in HCV-positive HCC patients based on genome sequencing profiling [45]. A recent study uncovered a cis-activating mechanism of host *ELF4* and HBV integration at the TERT promoter, which might result in TERT activation in HCC pathogenesis [46]. However, tumorigenesis is a complex and multistep process involving various molecules, and the significance of *ELF4* fusion in liver cancer occurrence remains to be clarified. Combined with our results, we assume that ELF4 might be a potential prognostic immune-related gene in CHOL.

Our study has some limitations. The number of CHOL samples derived from TCGA or GEO databases was small because CHOL is a rare cancer type, and our results might be biased. Second, the mechanisms of prognostic immune-related genes, such as lncRNA MIR99AHG, hsa-miR-25-3p, *ELF4*, and *LDHD*, in the pathogenesis and progression of CHOL require verification by more experiments *in vitro* and *in vivo*.

## 5. Conclusions

In conclusion, based on ceRNA network analysis, we identified that *lncRNA-MIR99AHG* may regulate the progression of CHOL by interacting with *hsa-miR-25-3p*. The ten-signature gene model provided an effective and reliable method for prognostic prediction in patients with CHOL. *ELF4*, *ABCG2*, *AGXT*, *LDHD*, *CKAP2L*, *LZTS1*, *PARPBP*, and *C6* may be prognostic immune-related genes that modulate the subsequent development of CHOL. Our findings would improve our understanding of the progression and recurrence of CHOL and lay the foundation for potential biomarkers or targets for the diagnosis and treatment of patients with CHOL.

## Figures and Tables

**Table 1:** Preservation analysis to identify critical gene modules.

ID	Color	Module size	Preservation information	
			*Z*-score	*P* value
Module 1	Blue	231	9.7356	4.70*E*-23
Module 2	Brown	88	10.9180	5.80*E*-20
Module 3	Green	27	1.1596	3.20*E*-01
Module 4	Grey	279	8.8745	1.90*E*-23
Module 5	Red	23	1.5823	5.90*E*-02
Module 6	Turquoise	284	30.7275	2.30*E*-19
Module 7	Yellow	60	7.6647	1.30*E*-07

The *Z*-score represents the stability of gene modules. Generally, modules with 5 a Z score < 10 were interpreted as stable modules, whereas those with *Z* scores > 10 were defined as highly stable. The *P* value indicates a significant correlation of modules.

**Table 2:** GO terms and KEGG signaling pathways analysis for candidate genes in ceRNA regulatory network.

Category	Term	Count	*P* value
Biology process	GO:0006865~amino acid transport	3	1.18*E*-02
	GO:0043065~positive regulation of apoptotic process	6	1.35*E*-02
	GO:0061304~retinal blood vessel morphogenesis	2	1.87*E*-02
	GO:0007155~cell adhesion	7	2.10*E*-02
	GO:0009404~toxin metabolic process	2	2.79*E*-02
	GO:0030574~collagen catabolic process	3	3.65*E*-02
	GO:0032835~glomerulus development	2	4.16*E*-02
	GO:0060068~vagina development	2	4.16*E*-02
	GO:0043547~positive regulation of GTPase activity	7	4.99*E*-02
	GO:0048016~inositol phosphate-mediated signaling	2	4.51*E*-02
	GO:0048514~blood vessel morphogenesis	2	4.77*E*-02
	GO:1990138~neuron projection extension	2	4.86*E*-02

KEGG pathway	hsa04974: protein digestion and absorption	4	1.24*E*-02
	hsa04512: ECM-receptor interaction	3	4.83*E*-02
	hsa05205: proteoglycans in cancer	4	4.97*E*-02

**Table 3:** The correlation between clinical features and the prognosis of cholangiocarcinoma patients using univariate and multivariate cox regression analysis.

Clinical characteristics	TCGA (*N* = 38)	Univariable Cox		Multivariable Cox	
		HR (95% CI)	*P* value	HR (95% CI)	*P* value
Age (years, mean ± SD)	63.03 ± 12.85	1.009 [0.973-1.048]	6.21*E*-01	1.076 [1.001-1.157]	4.65*E*-02
Gender (male/female)	16/20	1.387 [0.544-3.534]	4.92*E*-01	1.516 [0.314-7.314]	6.04*E*-01
Neoplasm histologic grade (G1/G2/G3/G4)	1/15/18/2	1.096 [0.412-2.407]	9.93*E*-01	1.069 [0.266-4.307]	9.25*E*-01
Pathologic M (M0/M1/-)	28/5/3	1.650 [0.462-5.891]	4.36*E*-01	0.140 [0.005-3.914]	2.47*E*-01
Pathologic N (N0/N1/-)	26/5/5	2.289 [0.602-8.700]	2.12*E*-01	0.031 [0.0003-3.108]	1.39*E*-01
Pathologic T (T1/T2/T3)	19/12/5	1.209 [0.666-2.196]	5.40*E*-01	0.120 [0.003-4.166]	2.42*E*-01
Pathologic stage (I/II/III/IV)	19/9/1/7	1.327 [0.923-1.907]	1.19*E*-01	21.49 [0.669-69.62]	8.31*E*-02
Prognostic score status (high/low)	18/18	6.074 [1.926-19.15]	5.65*E*-04	5.089 [1.770-33.653]	1.91*E*-02

SD: standard deviation.

**Table 4:** Multivariate Cox regression analysis of differential expressed RNAs correlated with cholangiocarcinoma prognosis.

ID	Type	Coefficient	Pr (>|*z*|)	Hazard ratio	95% CI	Upregulation
MIR99AHG	lncRNA	0.51439	0.001859	1.673	1.210-2.313	Down
MIR25	miRNA	0.04432	0.007761	1.045	1.012-1.080	Up
ELF4	mRNA	-0.07212	0.000645	0.930	0.893-0.970	Up
LDHD	mRNA	0.09162	0.004819	1.096	1.028-1.168	Down
C6	mRNA	0.02811	0.016096	1.029	1.005-1.052	Down
AGXT	mRNA	-0.04822	0.018002	0.953	0.916-0.992	Down
CKAP2L	mRNA	0.07573	0.02636	1.079	1.009-1.153	Up
ABCG2	mRNA	0.10740	0.029302	1.113	1.011-1.226	Down
LZTS1	mRNA	-0.08410	0.030723	0.919	0.852-0.992	Up
PARPBP	mRNA	0.14498	0.031683	1.156	1.013-1.320	Up

## Data Availability

Data sharing is not applicable to this article as no new data were created or analyzed in this study.

## Abbreviations

CHOL: Cholangiocarcinoma
HCC: Hepatocellular carcinoma
DERs: Differentially expressed RNAs
lncRNA: Long noncoding RNAs
ceRNA: Competing endogenous RNA
log2 FC: log2 fold change
PS: Prognostic score
WGCNA: Weighted gene coexpression network analysis.

## Supplementary Materials

Supplementary Materials Supplementary table S1 shows the sequences of the primers.
