# Supplementary material for: Identification of Prognostic Factors in Cholangiocarcinoma Based on Integrated ceRNA Network Analysis
Source: Comput Math Methods Med. 2022 Sep 15;2022:7102736. doi: 10.1155/2022/7102736 (PMC9499749; doi:10.1155/2022/7102736)
Supplement: Supplementary Materials — Supplementary table S1 shows the sequences of the primers. [file 7102736.f1.docx]

Supplementary table S1. The sequences of all primers.

| **Primers** | **Sequence (5’-3’)** |
| --- | --- |
| universal primer | GTGCAGGGTCCGAGGT |
| hsa-U6-RT | GTCGTATCCAGTGCAGGGTCCGAGGTATTCGCACTGGATACGACAAAATATG |
| hsa-U6-F | CTCGCTTCGGCAGCACA |
| hsa-U6-R | AACGCTTCACGAATTTGCGT |
| hsa-miR-25-3p-JH | GTCGTATCCAGTGCAGGGTCCGAGGTATTCGCACTGGATACGACTCAGAC |
| hsa-miR-25-3p-F | GCGCCATTGCACTTGTCTCG |
| ELF4-hF | CCTGATCTTTGAGTTCGCAAGC |
| ELF4-hR | AGTCCCGAGTACAGATGCAGT |
| CKAP2L-hF | GAGCCAAAACACCAAGCCTTA |
| CKAP2L-hR | GGAGTTTAATGCTGATGGACCTT |
| LZTS1-hF | AGCGTCAGTAGCCTCATCTC |
| LZTS1-hR | AGTCTTCGCTCTTGCCCATTT |
| PARPBP-hF | AATTGGCGTGCTCTTTGTAACT |
| PARPBP-hR | TCAGTCACGTCCATGTTTTCAA |
| LDHD-hF | AGGTGCGAACCTCCTGATG |
| LDHD-hR | CGGTGCCGAATGGGATGAT |
| C6-hF | TTGATGGGCAATGGGTTTCAT |
| C6-hR | ACTTGTCCTACTGCTTTTGACAG |
| AGXT-hF | CACCCGATGACCAAGGACC |
| AGXT-hR | CTCCCCGTGGGTTAAGAACAG |
| ABCG2-hF | ACGAACGGATTAACAGGGTCA |
| ABCG2-hR | CTCCAGACACACCACGGAT |
| MIR99AHG-hF | TTTGCTTCAAACGACAACAAGAG |
| MIR99AHG-hR | TTCGAGCATGGCCGGATTG |
| GAPDH-hF | GAGCTGCAATCGAAGTCTGG |
| GAPDH-hR | AAGGCCTTCTGTGAGTCCTC |
